# Orientational Fluctuations and Bimodality in Semiflexible Nunchucks

**DOI:** 10.3390/polym13122031

**Published:** 2021-06-21

**Authors:** Panayotis Benetatos, Mohammadhosein Razbin

**Affiliations:** 1 Department of Physics, Kyungpook National University, 80 Daehakro, Bukgu, Daegu 41566, Korea; 2 Department of Energy Engineering and Physics, Amirkabir University of Technology, Tehran 14588, Iran

**Keywords:** wormlike chain, hinged polymers, conformations, tensile elasticity, bimodality

## Abstract

Semiflexible nunchucks are block copolymers consisting of two long blocks with high bending rigidity jointed by a short block of lower bending stiffness. Recently, the DNA nanotube nunchuck was introduced as a simple nanoinstrument that mechanically magnifies the bending angle of short double-stranded (ds) DNA and allows its measurement in a straightforward way [Fygenson et al., Nano Lett. 2020, 20, 2, 1388–1395]. It comprises two long DNA nanotubes linked by a dsDNA segment, which acts as a hinge. The semiflexible nunchuck geometry also appears in dsDNA with a hinge defect (e.g., a quenched denaturation bubble or a nick), and in end-linked stiff filaments. In this article, we theoretically investigate various aspects of the conformations and the tensile elasticity of semiflexible nunchucks. We analytically calculate the distribution of bending fluctuations of a wormlike chain (WLC) consisting of three blocks with different bending stiffness. For a system of two weakly bending WLCs end-jointed by a rigid kink, with one end grafted, we calculate the distribution of positional fluctuations of the free end. For a system of two weakly bending WLCs end-jointed by a hinge modeled as harmonic bending spring, with one end grafted, we calculate the positional fluctuations of the free end. We show that, under certain conditions, there is a pronounced bimodality in the transverse fluctuations of the free end. For a semiflexible nunchuck under tension, under certain conditions, there is bimodality in the extension as a function of the hinge position. We also show how steric repulsion affects the bending fluctuations of a rigid-rod nunchuck.

## 1. Introduction

Semiflexible polymers are macromolecules with a finite bending stiffness. Some of the most important biomolecules, such as DNA or the structural elements of the cytoskeleton (F-actin, microtubules, intermediate filaments), are natural semiflexible polymers [1]. DNA nanotudes or carbon nanotubes are synthetic ones. A widely used minimal theoretical model of semiflexible polymers is the wormlike chain model (WLC), which represents the polymer by a one-dimensional, locally inextensible, fluctuating curve with bending stiffness [2,3]. The physical properties of the WLC are determined by the interplay of bending energy and conformational entropy [4].

Bending is a defining property of semiflexible polymers, and its measurement is an experimental task of central importance. In the case of double-stranded (ds) DNA, various techniques have been used in order to measure the bending angle, ranging from gel shift electrophoresis [5] to atomic force microscopy [6]. Recently, Fygenson et al. introduced a novel physical method to measure the bending angle with minimal sample preparation, direct visualization, and straightforward analysis procedures [7,8]. It involves the assembly of semiflexible “nunchucks”, composed of two long DNA nanotubes of large bending stiffness end-linked by a short segment of dsDNA, which acts as a hinge. The whole structure is confined between two glass plates. The fluctuations of the two arms are visualized through fluorescent video microscopy. The nunchuck arrangement effectively magnifies the bending fluctuations of the linking dsDNA segment.

Besides the dsDNA bending measuring nanodevice, the nunchuck geometry appears in several other cases. Defects in dsDNA, such as denaturation bubbles (regions where the two strands separate) [9,10,11] or nicks (discontinuities of the double stranded structure, exposing a single strand), can be viewed as hinges facilitating bending. dsDNA is much stiffer to bending than ssDNA. Assuming a hinge defect with a long lifetime (quenched), the nunchuck geometry is a coarse representation of dsDNA with such a defect. The semiflexible nunchuck may also be viewed as an elementary structural element of end-linked stiff polymer networks. Another realization of the nunchuck geometry would be a pair of end-linked F-actin filamnets. Cross-linked F-actin filaments with very short dangling ends effectively fall in this category. The bending stiffness of the nunchuck link, in the case of DNA nanotubes or dsDNA, can be controlled by the length of the linking segment or defect (bubble, nick), respectively. In the case of F-actin, there is a plethora of actin binding proteins (ABPs) with different bending stiffness [12,13].

In this article, we analytically investigate various aspects of the conformations of semiflexible nunchucks. It is organized as folllows. In Section 1, we present some general theoretical results for the conformations of a wormlike chain in two dimensions. We obtain the distribution of bending fluctuations both for a uniform chain and for a chain consisting of three parts, each with a different bending stiffness. The latter is a theoretical model of a semiflexible nunchuck. In the stiff (weakly bending) limit, we present the joint distribution of positional-orientational fluctuations of a uniform chain. In Section 2, we derive the conformational probabilities of the free end for a grafted system of two weakly bending semiflexible chains end-linked together for two cases. First, we consider a stiff link forming a kink. Then we consider an aligning link (hinge), which acts as an orientational harmonic spring. We show that, under certain conditions, there is a pronounced bimodality in the distribution of transverse positional fluctuations. In Section 3, we discuss how steric repulsion (excluded volume interaction) between the two arms of a nunchuck with stiff arms would change the distribution of bending fluctuations. In Section 4, we consider the response of a semiflexible nunchuck to a tensile force applied at its ends. We show that, under certain conditions, there is bimodality in the response as a function of the position of the hinge position: stiffening, softening, and stiffening. We summarize and conclude in Section 5.

## 2. The Theoretical Model

### 2.1. The Orientational Fluctuations of a Single Grafted Chain

**Figure 1:**
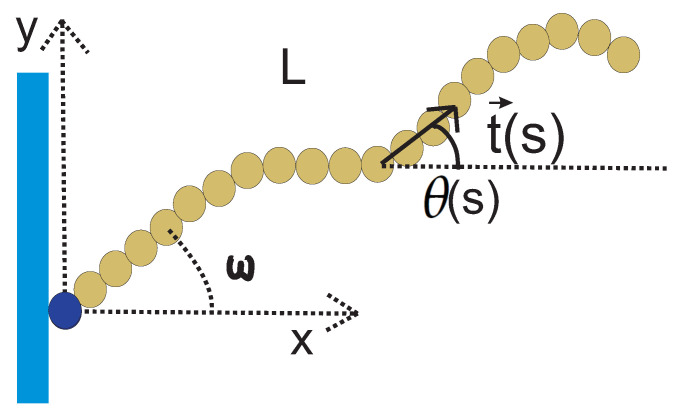
A typical configuration of a rather stiff grafted semiflexible filament. The persistence length of the filament is $l_{p}$ and it has contour length of $L<l_{p}$. The filament is grafted in a substrate with grafting angle, ω.

We consider a semiflexible filament, modelled as a wormlike chain (WLC), in the geometry shown in Figure 1. The total contour length of the chain is *L*, its persistence length is $l_{p}$, one of its ends is grafted (i.e., both its position and its orientation are fixed at that point), and it is confined to fluctuate in two dimensions. We point out that the grafting substrate shown in the figure is introduced solely for the purpose of specifying the geometry, and it does not imply any steric interaction with the chain (it can be viewed as a phantom substrate). The effective free energy functional of the WLC is

(1) $$H=\frac{\kappa }{2}\int_{0}^{L}ds{\left(\frac{\partial t(s)}{\partial s}\right)}^{2}=\frac{\kappa }{2}\int_{0}^{L}ds{\left(\frac{\partial \theta (s)}{\partial s}\right)}^{2}\;$$

where κ, t, and *s*, are the bending stiffness, the unit tangent vector and the arc length position of the filament, respectively. In two dimensions, $l_{p}=\frac{2\kappa }{k_{B}T}$. θ(s) is the angle that the tangent vector at *s* makes with the *x*-axis. The path θ(s), for 0<s<L, uniquely determines the conformation of the WLC in two dimensions.

The orientational fluctuations of the tangent vector at arc length position *s* are given by the conditional probability density to find it in direction t, given that at s=0, it points in $t_{0}$,

(2) $$P\left(t|t_{0}\right)=N\int_{t\left(0\right)=t_{0}}^{t(s)=t}D\left[t\left(s^{\prime }\right)\right]\delta (|t\left(s^{\prime }\right)\left|-1\right)\exp\left(-\frac{\kappa }{2k_{B}T}\int_{0}^{L}ds^{\prime }{\left(\frac{\partial t(s^{\prime })}{\partial s^{\prime }}\right)}^{2}\right)\;$$

where N is a normalization prefactor. $D[t\left(s^{\prime }\right)]$ denotes functional integration over all conformations of the tangent vector $t(s^{\prime })$. The condition |t(s)|=1 expresses the local inextensibility constraint of the WLC. The functional integral of Equation (2) is formally identical to that of the density operator of a two-dimensional rigid rotator of moment of inertia *I* [14,15,16]. The analogy is based on the following mapping: κ↔I, $k_{B}T↔\hbar$ and $s↔\hbar /(k_{B}\tilde{T})$ (where $\tilde{T}$ is the temperature of the quantum mechanical rotator). It is known that the density operator in this case obeys an imaginary-time Schrödinger equation,

(3) $$\hbar \frac{\partial P}{\partial s}=\frac{\hbar^{2}}{2I}\frac{\partial^{2}P}{\partial \theta^{2}}⟷\frac{\partial P}{\partial s}=\frac{k_{B}T}{2\kappa }\frac{\partial^{2}P}{\partial \theta^{2}}\;$$

In quantum mechanics, the state of the rotator (in the absence of internal degrees of freedom) is invariant under a full rotation by 2π. Thus, the wavefunction and the density operator are expressed in terms of the angular momentum eigenfunctions [17],

(4) $$|m\rangle =\frac{1}{\sqrt{2\pi }}e^{im\theta },\;\;\;\;m=0,\pm 1,\pm 2,...$$

and the solution to Equation (3) is

(5) $$P\left(t|t_{0}\right)=\frac{1}{2\pi }\sum_{m=-\infty }^{\infty }e^{-m^{2}\frac{s}{l_{p}}}e^{im(\theta -\omega )}=\frac{1}{2\pi }\vartheta_{3}\left(\frac{\theta -\omega }{2},\;\;e^{-\frac{s}{l_{p}}}\right)\;$$

where ω=θ(0) is the initial orientation (in the polymer case, the orientation of the grafted end) and $\vartheta_{3}\left(u,q\right)=\sum_{m=-\infty }^{\infty }q^{m^{2}}e^{2mui}$ is the Jacobi theta function of third order. It is clear that the solution to $P(t|t_{0})$ given in Equation (5) as a function of θ has a periodicity of 2π. Quite often, this result is directly transferred from the quantum rotator to the WLC [18,19,20,21,22]. However, this mathematical analogy should not be carried too far. The two-point orientational probability distribution of the WLC, unlike the quantum rotator density operator or propagator, is not invariant under full rotations of the tangent vector at a point along the chain contour. Such a rotation corresponds to a different conformation of the polymer. Indeed, this point becomes clear in the analysis of loops in Euler elastica [23,24]. In our case, the solution to Equation (3) belongs to a different functional space from that of the quantum rotator states and should be expressed in terms of “plane waves”,

(6) $$|k\rangle =\frac{1}{\sqrt{2\pi }}e^{ik\theta },\;\;\;\;-\infty <k<\infty \;$$

so that

(7) $$P\left(t|t_{0}\right)=\frac{1}{2\pi }\int_{k=-\infty }^{\infty }dk\;e^{-\frac{k^{2}s}{l_{p}}}\;e^{ik(\theta -\omega )}=\sqrt{\frac{l_{p}}{4\pi s}}\exp\left(-\frac{{\left(\theta -\omega \right)}^{2}l_{p}}{4s}\right)\;$$

**Figure 2:**
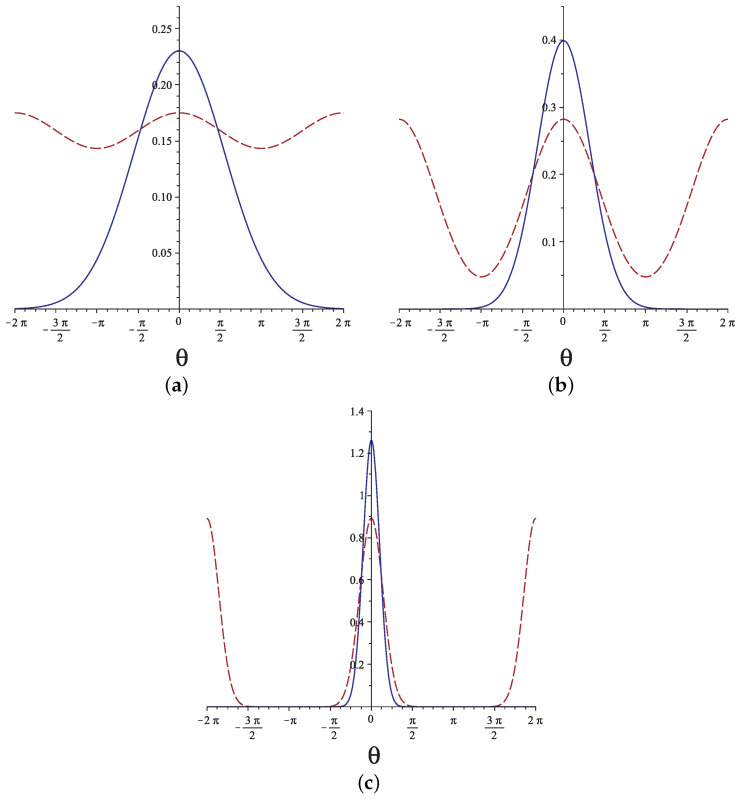
The probability of θ(L) (assuming ω=0) from the Gaussian solution (solid lines) and from the quantum analogy (dashed lines) for (**a**) $L/l_{p}=3$, (**b**) $L/l_{p}=1$, and (**c**) $L/l_{p}=0.1$.

This is a Gaussian with average 〈θ(s)〉=ω and standard deviation $\sqrt{\langle {\left(\theta \left(s\right)-\omega \right)}^{2}\rangle }=2s/l_{p}$. In Figure 2, we plot the orientational probability of the free end of a grafted chain calculated from Equation (7) (solid line) and from Equation (5) (dashed line) for $L/l_{p}=3,1,\;0.1$. We see that, except for very stiff chains and ignoring very large fluctuations, the discrepancy between the two approaches is quite significant.

For a WLC in three dimensions, it is customary to expand the corresponding two-point orientational probability density in terms of spherical harmonics, but this expansion—which is correct for a quantum particle on the unit sphere—suffers from the same symmetry problems that we described above [3]. However, unlike its two-dimensional counterpart, the resolution of this problem is much more challenging [25].

### 2.2. The Orientational Propagator and the Fluctuations of a Semiflexible Nunchuck

The Gaussian two-point orientational probability distribution of Equation (7) is in fact the Green’s function or propagator of Equation (3). If the probability density of the tangent vector orientation at point *s* is P(θ,s), we can find the probability density of the tangent vector orientation at point $s^{\prime }>s$ through integration:

(8) $$P\left(\theta^{\prime },s^{\prime }\right)=\int_{-\infty }^{\infty }\;d\theta \;P\left(\theta^{\prime },s^{\prime }|\theta ,s\right)P\left(\theta ,s\right)\;$$

where

(9) $$P\left(\theta^{\prime },s^{\prime }|\theta ,s\right)=\sqrt{\frac{l_{p}}{4\pi (s^{\prime }-s)}}\exp\left[-\frac{l_{p}{\left(\theta^{\prime }-\theta \right)}^{2}}{4(s^{\prime }-s)}\right]\;$$

This propagator is particularly useful in the analysis of the bending fluctuations of DNA nunchucks. Fygenson et al. [7] attached both ends of short double-stranded DNA (dsDNA) chains to fluorescently labeled DNA nanotubes confined to fluctuate in two dimensions. They extracted the distribution of the bending angle of the dsDNA through direct imaging of the nanotubes. This “nunchuck” arrangement can be used as an instrument to characterize the properties of dsDNA according to the WLC model (bending stiffness, contour length). The nunchuck can be viewed as a semiflexible block copolymer consisting of three blocks: the two DNA nanotubes and the dsDNA bridge. The attachment of the dsDNA is such that the tangent vector varies smoothly without any kink along the three blocks.

Let us consider the most general case of a nanotube of contour length $L_{1}$ and persistence length $l_{p1}$ connected to a nanotube of contour length $L_{3}$ and persistence length $l_{p3}$ connected through a dsDNA segment of contour length $L_{2}$ and persistence length $l_{p2}$. Using the orientational propagator, we can calculate the probability density of the fluctuations of the tangent vectors at the two free ends:

(10) $$\begin{array}{cc} P(\theta |\omega ) & =\int_{-\infty }^{\infty }\int_{-\infty }^{\infty }\;d\theta_{1}\;d\theta_{2}\;P\left(\theta ,L_{3}|\theta_{2},0\right)\;P\left(\theta_{2},L_{2}|\theta_{1},0\right)\;P\left(\theta_{1},L_{1}|\omega ,0\right)\\ \; & =\sqrt{\frac{1}{\left(4\pi \right)\left(L_{1}/l_{p1}+L_{2}/l_{p2}+L_{3}/l_{p3}\right)}}\exp\left[-\frac{{\left(\theta -\omega \right)}^{2}}{4(L_{1}/l_{p1}+L_{2}/l_{p2}+L_{3}/l_{p3})}\right] \end{array}$$

To connect Equation (10) with Reference [7], the middle segment of contour length $L_{2}$ corresponds to the dsDNA chain. In the experiment, its elasticity is probed by measuring the bending angle $\theta_{1}-\theta_{2}$. Equation (10) and the propagator method in general provide complementary constraints which can be used in the measurement of the bending stiffness of the middle chain. We point out that these expressions are exact, provided that all the chains can be described by the WLC model and there are no collisions (excluded volume) of chain segments.

### 2.3. The Positional-Orientational Propagator of a Wormlike Chain in the Stiff Limit

In this subsection, we consider a grafted WLC (in two dimensions, as shown in Figure 1) in the weakly bending stiff limit. Because of the large value of the bending rigidity, $L\ll l_{P}$, and since the deflection away from the grafting direction is small, sin(θ−ω)≈θ−ω and cos(θ−ω)≈1. The positional-orientational propagator of the chain is denoted by $G_{L,l_{p}}\left(x,y,\theta |x_{0},y_{0},\omega \right)$ and can be interpreted as the conditional probability density to find the endpoint of the chain at position (x,y) with orientation θ given that it is grafted at position $\left(x_{0},y_{0}\right)$ with orientation ω.

In the weakly bending regime, the propagator is calculated as a closed analytic expression [26,27,28,29,30]

(11) $$\begin{array}{c} G_{L,l_{p}}\left(x,y,\theta |x_{0},y_{0},\omega \right)=\frac{1}{N_{G}}exp[-\frac{3l_{p}}{L^{3}}(\left(y-y_{0}\right)\cos\left(\omega \right)\\ -\left(x-x_{0}\right)\sin\left(\omega \right){)}^{2}-\frac{l_{p}}{L}{\left(\theta -\omega \right)}^{2}]\\ \times exp[\frac{3l_{p}}{L^{2}}\left(\left(y-y_{0}\right)\cos\left(\omega \right)-\left(x-x_{0}\right)\sin\left(\omega \right)\right)\left(\theta -\omega \right)]\\ \times \delta [\left(x-x_{0}\right)\cos\left(\omega \right)+\left(y-y_{0}\right)\sin\left(\omega \right)-L]\; \end{array}$$

where δ(x) is the Dirac δ-function and the factor $N_{G}$ is determined by the normalization condition,

(12) $$\begin{array}{c} \int \int \int dxdyd\theta \;G_{L,l_{p}}\left(x,y,\theta |x_{0},y_{0},\omega \right)=1\; \end{array}$$

In the remainder of this article, we use the notation $\int \equiv \int_{-\infty }^{+\infty }$ for the sake of simplicity. Using Equation (11), we can easily calculate the probability density of the the *x* component of the free endpoint position,

(13) $$\begin{array}{cc} P_{x}\left(x\right) & =\int \int dyd\theta G_{L,l_{p}}\left(x,y,\theta |0,0,\omega \right)\\ & =\sqrt{\frac{3l_{p}}{4\pi L^{3}\sin^{2}\left(\omega \right)}}\exp\left(-\frac{3l_{p}{\left(x-L\cos\left(\omega \right)\right)}^{2}}{4L^{3}\sin^{2}\left(\omega \right)}\right)\; \end{array}$$

The probability density of the the *y* component of the free endpoint position is

(14) $$\begin{array}{cc} P_{y}\left(y\right) & =\int \int dxd\theta G_{L,l_{p}}\left(x,y,\theta |0,0,\omega \right)\\ & =\sqrt{\frac{3l_{p}}{4\pi L^{3}\cos^{2}\left(\omega \right)}}\exp\left(-\frac{3l_{p}{\left(y-L\sin\left(\omega \right)\right)}^{2}}{4L^{3}\cos^{2}\left(\omega \right)}\right)\; \end{array}$$

In addition, the probability density of the orientation of the free endpoint is given by

(15) $$\begin{array}{cc} P_{\omega }\left(\theta \right) & =\int \int dxdyG_{L,l_{p}}\left(x,y,\theta |0,0,\omega \right)\\ & =\sqrt{\frac{l_{p}}{4\pi L}}\exp\left(-\frac{l_{p}{\left(\theta -\omega \right)}^{2}}{4L}\right)\; \end{array}$$

We point out that even though Equations (13) and (14) rely on the validity of the weakly bending approximation, Equation (15) is exact.

## 3. Conformational Probabilities of Kinked and Hinged Stiff Chains

### 3.1. Positional Fluctuations of Two Weakly Bending WLCs Jointed at a Stiff Kink Point

**Figure 3:**
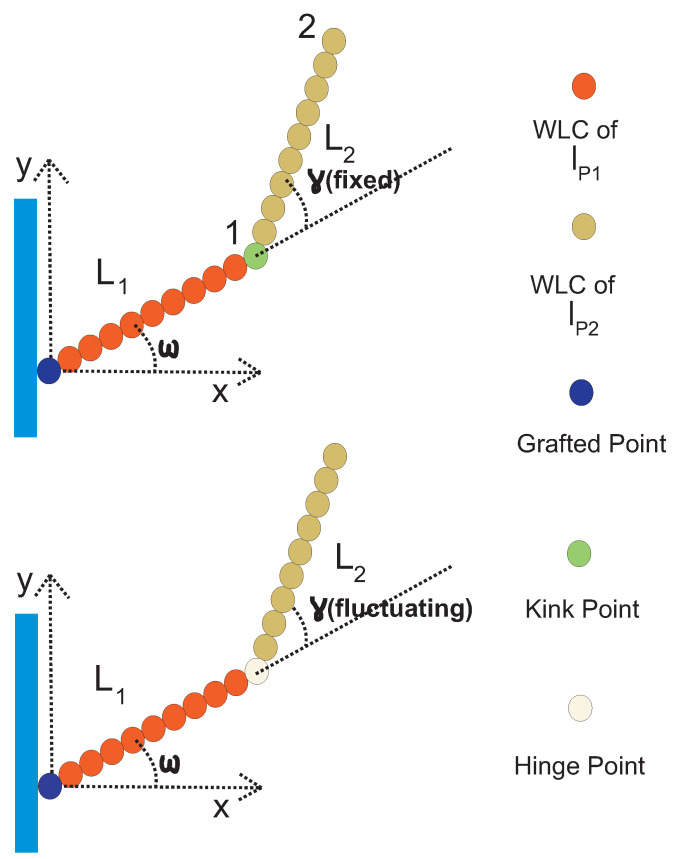
Upper panel: A configuration of two jointed weakly bending semiflexible filaments. The stiff joint (kink point) has a kink angle γ. The first filament has length $L_{1}$ and persistence length $l_{p1}$. The second filament has length $L_{2}$ and persistence length $l_{p2}$. The first filament is grafted on a substrate with a grafting angle ω. Lower panel: A configuration of two jointed semiflexible filaments with a hinged point. It differs from the system in the upper panel in that the kink angle γ fluctuates about an average value $\gamma_{0}$. The hinge point has a rotational stiffness $K_{h}$. For $K_{h}\to \infty$, the hinge point becomes a kink point and we recover the upper panel case.

We consider two WLCs, both in the stiff limit, which can have different persistence lengths. They are jointed at a kink, and the kink angle, γ, is fixed (it does not fluctuate). In Figure 3, we show the configuration of such a grafted kinked pair of arms. The first arm is grafted on the substrate with orientation ω and it has persistence length $l_{p1}$ and contour length $L_{1}$. The second arm is attached to the endpoint of the first arm at the kink point, and it has persistence length $l_{p2}$ and contour length, $L_{2}$. We label the endpoint of the first arm by the number 1, which is also the kink point. We also label the endpoint of the second arm of the structure by the number 2. The endpoint of the structure is the same as the endpoint of the second arm.

Concatenating the propagators associated with the two arms, we calculate the probability density to find the *x* component of the position of the free endpoint of the kinked structure at a given value $x_{2}$,

(16) $$\begin{array}{cc} P_{kx}\left(x_{2}\right) & =\int \int \int dx_{1}dy_{1}d\theta_{1}G_{L_{1},l_{p1}}\left(x_{1},y_{1},\theta_{1}|0,0,\omega \right)\\ & \times \int \int dy_{2}d\theta_{2}G_{L_{2},l_{p2}}\left(x_{2},y_{2},\theta_{2}|x_{1},y_{1},\theta_{1}+\gamma \right)\; \end{array}$$

Similarly, we calculate the probability density to find the *y* component of the position of the endpoint of the filament at $y_{2}$,

(17) $$\begin{array}{cc} P_{ky}\left(y_{2}\right) & =\int \int \int dx_{1}dy_{1}d\theta_{1}G_{L_{1},l_{p1}}\left(x_{1},y_{1},\theta_{1}|0,0,\omega \right)\\ & \times \int \int dx_{2}d\theta_{2}G_{L_{2},l_{p2}}\left(x_{2},y_{2},\theta_{2}|x_{1},y_{1},\theta_{1}+\gamma \right)\; \end{array}$$

By performing the integrals in Equation (16), we obtain an analytic expression for $P_{x}\left(x_{2}\right)$:

(18) $$P_{kx}\left(x_{2}\right)=\frac{1}{\sqrt{\pi }\sigma_{kx}}\exp\left(-\frac{{\left(x_{2}-L_{1}\cos\left(\omega \right)-L_{2}\cos\left(\omega +\gamma \right)\right)}^{2}}{{\sigma_{kx}}^{2}}\right)\;$$

where

(19) $$\begin{array}{ccc} {\sigma_{kx}}^{2} & = & \frac{4}{3l_{p1}l_{p2}}\left[l_{p1}L_{2}^{3}+3l_{p2}L_{1}L_{2}^{2}\right]\sin{\left(\omega +\gamma \right)}^{2}\\ & & +\frac{12}{3l_{p1}}L_{1}^{2}L_{2}\sin\left(\omega \right)\sin\left(\omega +\gamma \right)\\ & & +\frac{4}{3l_{p1}}L_{1}^{3}\sin{\left(\omega \right)}^{2}\; \end{array}$$

Similarly, the Gaussian integrals in Equation (17) yield an analytic expression for $P_{y}\left(y_{2}\right)$:

(20) $$P_{ky}\left(y_{2}\right)=\frac{1}{\sqrt{\pi }\sigma_{ky}}\exp\left(-\frac{{\left(y_{2}-L_{1}\sin\left(\omega \right)-L_{2}\sin\left(\omega +\gamma \right)\right)}^{2}}{{\sigma_{ky}}^{2}}\right)\;$$

where

(21) $$\begin{array}{ccc} {\sigma_{ky}}^{2} & = & \frac{4}{3l_{p1}l_{p2}}\left[l_{p1}L_{2}^{3}+3l_{p2}L_{1}L_{2}^{2}\right]\cos{\left(\omega +\gamma \right)}^{2}\\ & & +\frac{12}{3l_{p1}}L_{1}^{2}L_{2}\cos\left(\omega \right)\cos\left(\omega +\gamma \right)\\ & & +\frac{4}{3l_{p1}}L_{1}^{3}\cos{\left(\omega \right)}^{2}\; \end{array}$$

In the weakly bending approximation, all the integrals are Gaussian. As a consistency check, we look at two limiting cases. In the first case, γ=0, $L_{1}=L_{2}=\frac{L}{2}$ and $l_{p}=l_{p1}=l_{p2}$, while in the second case, $L_{1}=0$, γ=0 and $l_{p}=l_{p2}$. In both cases, the two probability density functions of Equations (18) and (20) reduce to Equations (13) and (14) respectively, which correspond to a single filament with length *L*.

### 3.2. Bimodality in the Positional Fluctuations of Two Weakly Bending WLCs Jointed at a Hinge Point

The transverse fluctuations of a stiff grafted WLC ($L\ll l_{p}$) are Gaussian. As we see from Equation (14), if we set ω=0, $P_{y}\left(y\right)$ is peaked at y=0 and the variance of *y* is $2L^{3}/\left(3l_{p}\right)$. It is known that, as the stiffness of the WLC (given by $l_{p}/L$) decreases, $P_{y}\left(y\right)$ changes from its original Gaussian form and develops a bimodality, which can be viewed as the hallmark of semiflexibility ($l_{p}/L\approx 1$) [22,30,31,32,33]. Bimodality has also been observed in molecular dynamics simulations of semiflexible polymers in two dimensions under shear flow [34]. In the opposite limit of a flexible chain ($L\gg l_{p}$), $P_{y}\left(y\right)$ becomes Gaussian again (corresponding to an ideal chain).

If we have a stiff grafted WLC with a hinge point along its contour, the form of the distribution of transverse fluctuations of the free end, $P_{y}\left(y\right)$, strongly depends on the stiffness of the hinge point. An infinitely stiff hinge corresponds to the absence of hinge, because it secures the continuity of the tangent vector. A completely soft hinge yields a WLC freely jointed to a grafted WLC. Let us first consider the case of two perfectly rigid rods jointed by a completely soft hinge (lower panel of Figure 3 with ω=0 and $l_{p1}=l_{p2}=\infty$). We have $y=L_{2}\sin\left(\gamma \right)$ and $P_{h}\left(\gamma \right)=1/\left(2\pi \right)$ for 0≤γ<2π. We obtain

(22) $$P_{y}\left(y\right)=P_{h}\left(\gamma \right)|\frac{d\gamma }{dy}|=\frac{1}{2\pi L_{2}}\frac{1}{\sqrt{1-{\left(y/L_{2}\right)}^{2}}}\;$$

We can see that, in this case, the probability density has a pronounced bimodality with two integrable divergences at $y=\pm L_{2}$. We can easily understand this result from the geometry of a circle. For stiff rods and a soft hinge, the free tip has equal probability to be on any point along a circle of radius $L_{2}$. Conformations with *y* close to the radius correspond to more points along the circle (higher probability) than those with y≈0.

We now consider a grafted kinked filament as the one discussed in the previous subsection and allow the kink angle to thermally fluctuate. The result is that of two filaments jointed via a hinge point. The probability density of the *x* component of the position of the endpoint of a hinged polymer is given by the following expression,

(23) $$\begin{array}{cc} P_{hx}\left(x_{2}\right) & =\int \int \int \int d\gamma dx_{1}dy_{1}d\theta_{1}G_{L_{1},l_{p1}}\left(x_{1},y_{1},\theta_{1}|0,0,\omega \right)\\ & \times \int \int dy_{2}d\theta_{2}G_{L_{2},l_{p2}}\left(x_{2},y_{2},\theta_{2}|x_{1},y_{1},\theta_{1}+\gamma \right)P_{h}\left(\gamma \right) \end{array}$$

which can be rewritten as follows

(24) $$\begin{array}{c} P_{hx}\left(x_{2}\right)=\int P_{h}\left(\gamma \right)P_{kx}\left(x_{2}\right)d\gamma \; \end{array}$$

where $P_{kx}\left(x_{2}\right)$ is given by Equation (18) and

(25) $$P_{h}\left(\gamma \right)=\sqrt{\frac{K_{h}}{2\pi }}\exp\left[-\frac{K_{h}{\left(\gamma -\gamma_{0}\right)}^{2}}{2}\right]\;$$

As we see from Equation (25), we treat the hinge as an angular spring. This may represent an F-actin cross-linking protein, which prescribes a finite angle $\gamma_{0}\neq 0$ (e.g., filamin), or it may be a coarse-grained version of a short dsDNA link of the two DNA nanotubes in the nunchuck complex of Reference [7]. In the latter case, $\gamma_{0}=0$ and comparing Equation (25) with Equation (9) we have $K_{h}=2l_{p}/s$, where *s* is the length of the link.

Similarly, the probability density of the *y* component of position of the endpoint of a grafted hinged filament is

(26) $$\begin{array}{cc} P_{hy}\left(y_{2}\right) & =\int \int \int \int d\gamma dx_{1}dy_{1}d\theta_{1}G_{L_{1},l_{p1}}\left(x_{1},y_{1},\theta_{1}|0,0,\omega \right)\\ & \times \int \int dx_{2}d\theta_{2}G_{L_{2},l_{p2}}\left(x_{2},y_{2},\theta_{2}|x_{1},y_{1},\theta_{1}+\gamma \right)P_{h}\left(\gamma \right)\; \end{array}$$

We can rewrite the Equation (26) as

(27) $$P_{hy}\left(y_{2}\right)=\int P_{h}\left(\gamma \right)P_{ky}\left(y_{2}\right)d\gamma \;$$

where $P_{ky}\left(y_{2}\right)$ is given by Equation (20).

**Figure 4:**
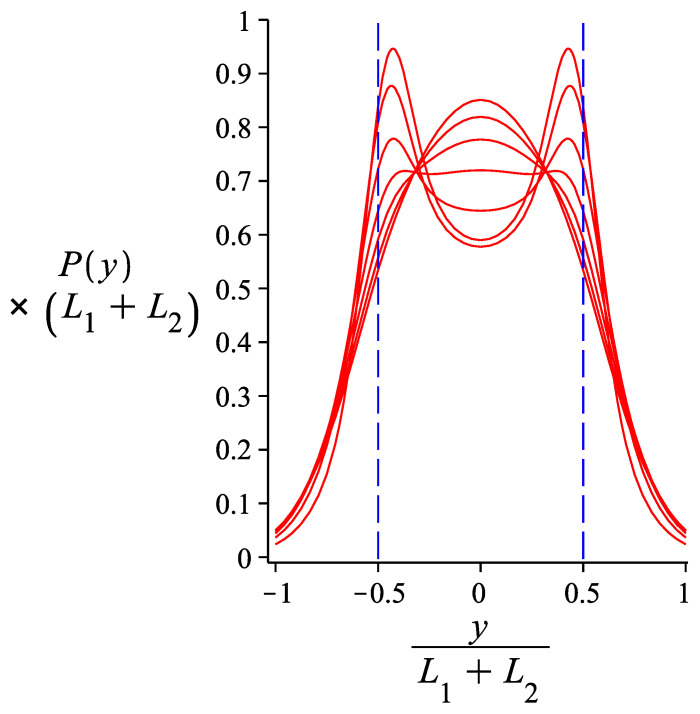
The probability density of the transverse component of the free end of a grafted system of two hinged WLCs. The curve is obtained from Equation (27). The varying parameter is the rotational stiffness of the hinge point (in units of the thermal energy, $k_{B}T$): $K_{h}=0.01+0.5\times i$ where i=0,1,2,3,4,5,6. The lower the hinge stiffness, the sharper the bimodality of the distribution. The fixed parameters are: $L_{1}=L_{2}=3\;\mu$m, $l_{p1}=l_{p2}=27\;\mu$m, ω=0.

**Figure 5:**
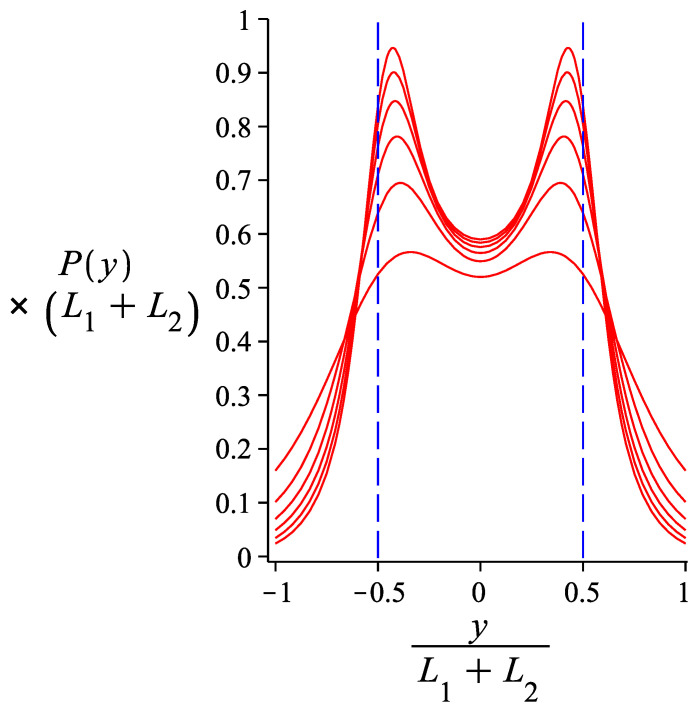
The probability density of the transverse component of the free end of a grafted system of two hinged WLCs. The curve is obtained from Equation (27). The varying parameter is $l_{p}$; $l_{p}\equiv l_{p1}=l_{p2}=4.5\times i\;\mu$m where i=1,2,3,4,5,6. The lower the value of $l_{p}$, the smoother the bimodality of the curves. The fixed parameters are: $L_{1}=L_{2}=3\;\mu$m, $\frac{K_{h}}{2}=0.005$ and ω=0.

We now focus on a hinge with $\gamma_{0}=0$ and calculate numerically the integrals that give $P_{hy}\left(y_{2}\right)$ in order to see the dependence of its form on the bending stiffness of the hinge and that of the two arms. In Figure 4, we use parameters appropriate for the DNA nanotube nunchuck experiments of Reference [7]. We assume that the two arms are identical, with contour length 3 μm and persistence length of 27 μm. We see that for a hinge strength of $K_{h}/\left(k_{B}T\right)=1.51$, which corresponds to a WLC link of $l_{p}/s=0.755$, the distribution of transverse fluctuations exhibits a plateau. For a hinge of higher stiffness, there is no bimodality (there is a single peak). For a hinge of lower stiffness, there is bimodality, which becomes more pronounced as the stiffness decreases. We see that the form of the distribution (flat, bimodal, unimodal) allows us to get a rough estimate of the stiffness of the link between the two arms of the nunchuck. In Figure 5, we see that for a link of given bending stiffness, a bimodal distribution of transverse fluctuations becomes less sharp as the stiffness of the nunchuck arms decreases.

In this Section, we focused on bimodality in the conformations of a nunchuck confined in two dimensions. Even though the three-dimensional case is beyond the scope of this article, we do not expect a qualitative case. This bimodality is similar to that in grafted wormlike chains, and Reference [33] shows that it persists in three dimensions.

## 4. Rigid-Rod Nunchuck with Excluded Volume Interaction

In our analysis so far, we have assumed that the system does not experience any excluded volume interaction. In Section 3.2, we integrate the hinge angle γ from −∞ to +∞, assuming that the two arms of the nunchuck can twist on top of each other. A more realistic approach would be to take into account the excluded volume interaction. Calculating conformational distributions with excluded volume interaction is a very challenging problem, which goes beyond the scope of the present article. However, we can do that for the simple case of a nunchuck that consists of two arms of infinite bending stiffness and a harmonic hinge. In that case, the conformations of the system are determined by the hinge angle, which is confined to fluctuate in the range −π<γ<π.

We consider the hinge as a WLC whose orientational fluctuations obey the diffusion-like equation, Equation (3). In Section 2.1, we showed that if the range of the bending angle is from −∞ to +∞, the distribution is Gaussian. If the range of fluctuations is restricted because of the excluded volume interaction of the nunchuck arms, we have to impose absorbing boundary conditions to the “diffusion” process [35]. We consider the geometry of Figure 1 with the “starting” angle ω=0. In order to solve Equation (3), we use a factorization ansatz,

(28) P(θ,s)=Q(θ)exp(−λs)

which yields the eigenfunction equation

(29) $$\left[\frac{d^{2}}{d\theta^{2}}+k^{2}\right]Q\left(\theta \right)=0\;,\;\;\;\;k^{2}=\frac{2\lambda \kappa }{k_{B}T}\;$$

The eigenfunctions of the harmonic oscillator are sines and cosines, and those consistent with the absorbing boundary conditions,

(30) Q(±π)=0

are cosines, $\cos(k_{n}\theta )$, with

(31) $$k_{n}=n+\frac{1}{2}\;,\;\;\;n=0,1,2,...$$

The solution of Equation (3) with absorbing boundary conditions reads

(32) $$P\left(\theta ,s\right)=N\sum_{n=0}^{\infty }cos\left(\frac{(2n+1)\theta }{2}\right)\exp\left(-\frac{{\left(2n+1\right)}^{2}s}{4l_{p}}\right)\;$$

where N is the normalization prefactor,

(33) $$N=4\sum_{n=0}^{\infty }\frac{{\left(-1\right)}^{n}}{2n+1}\exp\left(-\frac{{\left(2n+1\right)}^{2}s}{4l_{p}}\right)\;$$

**Figure 6:**
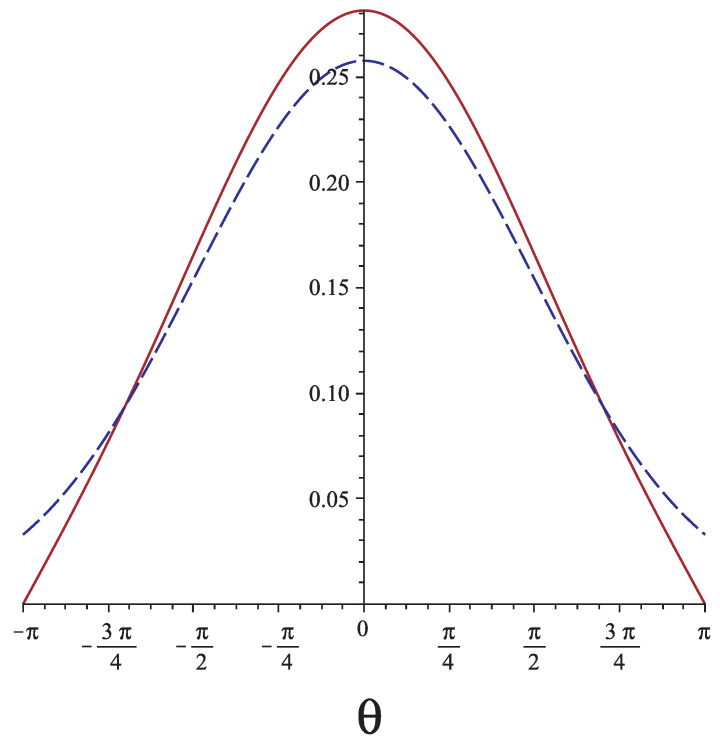
The probability density of the bending angle of a WLC hinge with ratio of contour length to persistent length $s/l_{p}=1.2$. The dashed line is the usual Gaussian, whereas the solid line is obtained by imposing absorbing boundary conditions to Equation (3).

The standard deviation of the hinge angle in the nunchucks of Reference [7] lies in the interval $\left[20^{\circ },83^{\circ }\right]$, which corresponds to a flexibility $s/l_{p}$ in the interval [0.174,0.724]. If we plot P(θ,s) as Gaussian from Equation (7) and as the solution of the diffusion equation with absorbing boundary conditions from Equation (32), we do not see any noticeable difference for $s/l_{p}=0.174$. For $s/l_{p}=0.724$, the discrepancy caused by the boundary conditions is small but noticeable. In Figure 6, we plot the bending distribution according to the usual Gaussian, Equation (7), against that of Equation (32) for $s/l_{p}=2$, and we see a significant discrepancy.

We point out that modeling the excluded volume interaction between the two rigid arms of the nunchuck via absorbing boundary conditions for the orientational distribution of the hinge WLC, P(θ,s), is an approximation even for infinitely long arms. The absorbing boundary conditions that we assume in order to obtain Equation (32) apply to the orientational conformations θ(s) over the entire contour length [0,s] of the WLC and not just the end point. Thus, they become unrealistic for a flexible hinge with $s/l_{p}\gg 1$. On the other hand, a flexible hinge would trivially yield a uniform distribution. Therefore, we conclude that Equation (32) is a good approximation for hinge links with $s/l_{p}≲1$.

## 5. Bimodality in the Tensile Elasticity of a Semiflexible Nunchuck

**Figure 7:**
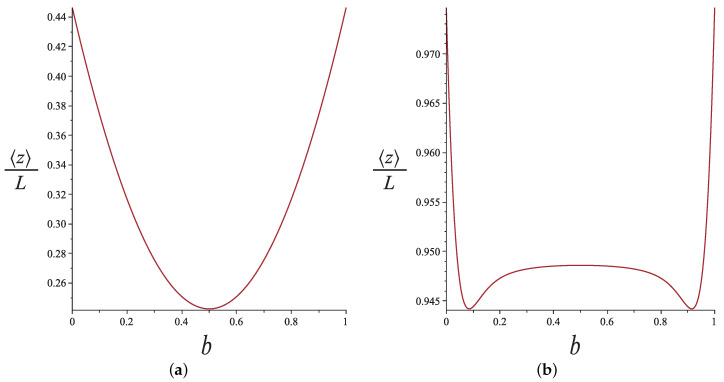
The average projection of the end-to-end distance n the direction of the force as a function of the hinge position for (**a**) $f=k_{B}T/L$ and (**b**) $f=20k_{B}T/L$

We consider a nunchuck in two dimensions, consisting of two rigid arms of length bL and (1−b)L, jointed by a fully flexible hinge and subjected to a tensile force f (0<b<1). The partition function reads

(34) $$\begin{array}{ccc} Z_{2d} & = & \int_{0}^{2\pi }\int_{0}^{2\pi }d\theta_{1}d\theta_{2}\exp\left(\frac{fbL}{k_{B}T}\cos\left(\theta_{1}\right)+\frac{f(1-b)L}{k_{B}T}\cos\left(\theta_{2}\right)\right)\\ & = & {\left(2\pi \right)}^{2}I_{0}\left(\frac{fbL}{k_{B}T}\right)I_{0}\left(\frac{f(1-b)L}{k_{B}T}\right)\; \end{array}$$

where $I_{n}$ is the modified Bessel function of the first kind of order *n*. The force-extension relation is obtained by taking the derivative,

(35) $$\langle z\rangle =k_{B}T\frac{\partial \ln(Z)}{\partial f}=Lb\frac{I_{1}\left(fbL/\left(k_{B}T\right)\right)}{I_{0}\left(fbL/\left(k_{B}T\right)\right)}+L\left(1-b\right)\frac{I_{1}\left(f\left(1-b\right)bL/\left(k_{B}T\right)\right)}{I_{0}\left(f\left(1-b\right)bL/\left(k_{B}T\right)\right)}\;$$

where 〈z〉 is the average end-to-end distance in the direction of the force. If we plot 〈z〉 as a function of the hinge position *b* as we do in Figure 7, we get a single minimum at the middle b=1/2 for small forces and a pronounced bimodality for higher forces. We can understand this as follows. For a strong tension, the long arm of the nunchuck is weakly tilting. On the other hand, the short arm reaches a length bL such that $bLf≲k_{B}T$ and fluctuates very strongly, causing the end-to-end distance to shrink before it gets back to its value corresponding to a single rod as b→0.

This should be contrasted with the elastic response in three dimensions. In that case, the partition function reads

(36) $$\begin{array}{cc} Z_{3d} & =\int_{0}^{2\pi }d\phi_{1}\int_{0}^{\pi }\sin\left(\theta_{1}\right)d\theta_{1}\int_{0}^{2\pi }d\phi_{2}\int_{0}^{\pi }\sin\left(\theta_{2}\right)d\theta_{2}\exp\left(\frac{fbL}{k_{B}T}\cos\left(\theta_{1}\right)+\frac{f(1-b)L}{k_{B}T}\cos\left(\theta_{2}\right)\right)\\ \; & ={\left(4\pi \right)}^{2}\frac{\sinh(fbL/\left(k_{B}T\right)}{fbL/(k_{B}T)}\frac{\sinh(f\left(1-b\right)L/\left(k_{B}T\right)}{f\left(1-b\right)bL/\left(k_{B}T\right)}\; \end{array}$$

The corresponding force-extension relation is

(37) $$\frac{\langle z\rangle }{L}=b\coth\left(\frac{fbL}{k_{B}T}\right)+\left(1-b\right)\coth\left(\frac{f(1-b)L}{k_{B}T}\right)-2\frac{k_{B}T}{fL}$$

If we plot 〈z〉 as a function of the hinge position *b*, we always get a convex function with a single minimum at the middle, b=0.5. We can rigorously confirm this by calculating its second derivative and checking its sign. The markedly different behavior from the two dimensional case is due to the extra space that is available to the strongly fluctuating small arm of the nunchuck. More specifically, for the weakly tilting long arm, we have decoupling of the two transverse dimensions. Thus, the corresponding contribution to the entropic elasticity is twice that of the two-dimensional case. On the other hand, the small segment responds linearly, and the corresponding tensile compliance in 3d is 3/2 times that in 2d. The increase in the fluctuations of the long arm overshadows the contribution from the short arm, and this results in a convex curve.

Let us now consider a nunchuck with semiflexible arms. In Reference [36], the force-extension relation of a semiflexible nunchuck with a hinge modeled as an orientational harmonic spring is calculated analytically, in the weakly bending approximation. (We point out that the weakly bending approximation may hold because of a strong tensile force or because of a large bending stiffness.) Adapting that result for a flexible hinge in two dimensions, we obtain

(38) $$\frac{\langle z\rangle }{L}=1-\frac{L}{l_{p}}\frac{1}{\tilde{f}}-\frac{1}{2}\frac{L}{l_{p}}\frac{\coth\left(\sqrt{\tilde{f}}b\right)\sqrt{\tilde{f}}b}{\tilde{f}}-\frac{1}{2}\frac{L}{l_{P}}\frac{\coth\left(\sqrt{\tilde{f}}\left(1-b\right)\right)\sqrt{\tilde{f}}\left(1-b\right)}{\tilde{f}}\;$$

where the dimensionless force is defined as $\tilde{f}=fL^{2}/\kappa$. It can also be written as $\tilde{f}={\left(L/l_{f}\right)}^{2}$ with $l_{f}=\sqrt{\kappa /f}$. This result is based on the force-extension relation of an intact WLC of contour length *L* with free hinged–hinged boundary conditions (in the weakly bending approximation) [19,36,37],

(39) $$\frac{\langle z\rangle }{L}=1-\frac{1}{2}\frac{L}{l_{P}}\frac{\coth\left(L/l_{f}\right)\left(L/l_{f}\right)+1}{{\left(L/l_{f}\right)}^{2}}\;$$

In order to understand the physical meaning of the tension-dependent characteristic length $l_{f}$, we compare the strong stretching limit of a WLC with that of a freely jointed chain (FJC) in two dimensions. To the leading order, the WLC yields

(40) $$\frac{\langle z\rangle }{L}=1-\frac{1}{4}\frac{k_{B}T}{\sqrt{\kappa f}}$$

while the FJC yields

(41) $$\frac{\langle z\rangle }{L}=1-\frac{k_{B}T}{2lf}\;$$

κ is the bending stiffness of the WLC, and *l* is the elementary segment (link) length of the FJC. Comparing the two expressions, we see that, under strong stretching, a WLC behaves as a FJC with a tension-dependent link length equal to $2l_{f}$. Thus $l_{f}$ plays the role of an orientational correlation length for a WLC under strong tension.

If we plot the extension of a hinged WLC under strong tension ($L\ll l_{f}$) from Equation (38) as a function of the hinge position, we see that it exhibits a plateau and decreases as the hinge approaches the end points. The decrease is significant within a contour distance $∼l_{f}$ from the end points. This is understood because the small segment at the end fluctuates strongly. However, there is a problem with this result. As *b* approaches 0 or 1, we should recover the result of the intact chain. The reason behind this apparent inconsistency is the breakdown of the weakly bending approximation for the small segment at the end. For a WLC with $L=l_{p}$ subject to tension, the extension given by Equation (39), which is based on the weakly bending approximation, vanishes at $l_{f}\approx L$, and it turns negative for smaller forces. In order to remedy this problem, we consider the small segment at the end of the nunchuck as a rigid rod freely jointed to the weakly bending WLC. We recall that the force-extension relation of freely rotating rigid rod of length *L* in two dimensions is

(42) $$\frac{\langle z\rangle }{L}=\frac{I_{1}\left(fL/\left(k_{B}T\right)\right)}{I_{0}\left(fL/\left(k_{B}T\right)\right)}\;$$

We point out that this is a general result and holds even beyond the weakly bending approximation. For a small segment at the end with length bL, the tensional energy becomes comparable to or smaller than the thermal energy, $bLf≲k_{B}T$, and that makes it fluctuate strongly. If we combine this with the force-extension relation of the weakly bending WLC of length (1−b)L, we obtain

(43) $$\frac{\langle z\rangle }{L}=b\frac{I_{1}\left(fbL/\left(k_{B}T\right)\right)}{I_{0}\left(fbL/\left(k_{B}T\right)\right)}+\left(1-b\right)-\frac{1}{2}\frac{L}{l_{P}}\frac{\coth\left(\sqrt{\tilde{f}}\left(1-b\right)\right)\sqrt{\tilde{f}}\left(1-b\right)+1}{\tilde{f}}\;$$

**Figure 8:**
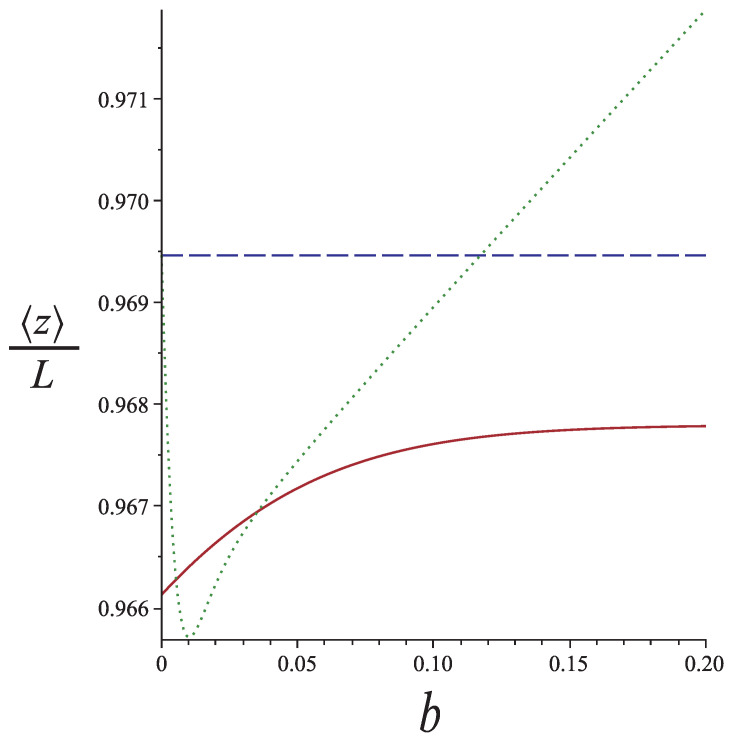
The mean extension of a semiflexible nunchuck in 2d as a function of the hinge position *b* for $l_{p}=L$ and $f=150k_{B}T/L$. The solid line corresponds to the weakly bending approximation of the hinged WLC (Equation (38)). The dotted line corresponds to a nunchuck whose small arm is a rigid rod and the long arm a weakly bending WLC (Equation (43)). The dashed line gives the extension of the corresponding intact WLC (without any hinge).

We can easily see that for b→0, we recover the extension of the intact WLC (without a hinge) with contour length *L*. The interesting feature of the extension as a function of the hinge position according to Equation (43) is that it has a minimum below the plateau of Equation (38) and that the minimum occurs at a value of *b* for which Equation (43) holds. Because the two ends are symmetric, we obtain bimodality in the response of a nunchuck to stretching as a function of the hinge position. Equation (43) holds for $bL≲l_{f}$, whereas Equation (38) holds for $bL≳2l_{f}$. Even though we do not have an exact result for the entire range of *b*, the bimodality is a robust feature. These features are shown in Figure 8. The tension and the bending stiffness of the arms correspond to $l_{f}\approx 0.058L$.

If we have a semiflexible nunchuck under tension in three dimensions, Equation (38) still holds, and the change in dimensionality only affects $l_{p}$ ($2\kappa /(k_{B}T)$ in 2d and $\kappa /(k_{B}T)$ in 3d). Equation (43), which gives the force–extension relation of a mixed nunchuck (rigid rod jointed to a semiflexible arm), will have a different first term,

(44) $$\frac{\langle z\rangle }{L}=b\coth\left(\frac{fbL}{k_{B}T}\right)-\frac{k_{B}T}{fL}+\left(1-b\right)-\frac{1}{2}\frac{L}{l_{P}}\frac{\coth\left(\sqrt{\tilde{f}}\left(1-b\right)\right)\sqrt{\tilde{f}}\left(1-b\right)+1}{\tilde{f}}\;$$

If we carry out the same analysis as we did for the 2d case, we see that the bimodality in the extension as a function of the hinge position persists, at least for $l_{p}\approx L$. As $l_{p}$ increases, the bimodality becomes less pronounced and eventually disappears because at the limit $l_{p}\to \infty$, we recover the rigid-arm nunchuck in 3d, described by Equation (7).

## 6. Conclusions

In this article, we theoretically investigated several aspects of the conformational and elastic behavior of the semiflexible nunchucks. Our results may help the analysis of experimental data. As some of our results have not been tested experimentally, they may motivate future experiments.

At first, we rigorously derive the distribution of bending fluctuations of a WLC in 2d and discuss the discrepancy with a widely used result, which is based on an imperfect analogy with a quantum rigid rotator. We point out that a similar analogy in 3d gives a propagator in terms of spherical harmonics [3], which is also widely used and suffers from the same problems (unjustified periodicity) as its counterpart in 2d. However, as opposed to the 2d case, there is no simple resolution to this problem [25]. Concatenating Gaussian propagators, we obtain the distribution of bending fluctuations of a three-block WLC comprising two long segments of high bending stiffness and a short segment of lower bending stiffness in the middle, which models a semiflexible nunchuck.

Using the positional-orientational propagator for the weakly bending approximation, which is valid for stiff WLCs, we analytically obtain the positional distribution of the free end of a pair of WLCs jointed by a rigid kink, assuming the other end to be grafted. If we replace the kink by an aligning hinge that acts a harmonic bending spring, we obtain (up to an integral that has to be calculated numerically) the probability of transverse fluctuations of the free end. We show that, depending of the stiffness of the hinge and the bending stiffness of the two arms, this distribution exhibits a pronounced bimodality, which gradually flattens and eventually vanishes as the hinge becomes stiffer. As the hinge can be viewed as a coarse-grained version of a short WLC linking the two arms of the nunchuck, the distinctive qualitative features of the distribution of transverse fluctuations (bimodal, flat, unimodal) could help us get a rough estimate of the stiffness of the hinge.

In most of this work, we ignore the excluded volume interaction. We assume that the two arms of the nunchuck are free to strongly fluctuate past each other. However, we also analyze the case of a nunchuck with rigid rod arms and excluded volume interaction. We show how the bending distribution changes from the Gaussian which is valid in the “phantom” case.

In the case of nunchucks with a soft (fully flexible) hinge, we analyze the response to a stretching force applied at the two ends. We are interested in the dependence of the nunchuck extension in the direction of the force on the hinge position. In the 2d case, if the two arms are rigid rods, we get a single minimum of the extension when the hinge is at the middle. However, for strong tension, we get bimodality. For a hinge close to the ends, we get two minima separated by a plateau. Interestingly, this bimodality (for a rigid-rod nunchuck) does not persist in 3d. For semiflexible nunchucks, at least when the total contour length of the intact WLC (without the hinge) is of the order of the persistence length, we obtain this bimodality under strong stretching, both in two and in three dimensions.

## Data Availability

Not applicable.
